# Enhanced Performance of GaN-based Ultraviolet Light Emitting Diodes by Photon Recycling Using Graphene Quantum Dots

**DOI:** 10.1038/s41598-017-07483-3

**Published:** 2017-08-02

**Authors:** Tzu-Neng Lin, Svette Reina Merden Santiago, Chi-Tsu Yuan, Kuo-Pin Chiu, Ji-Lin Shen, Ting-Chun Wang, Hao-Chung Kuo, Ching-Hsueh Chiu, Yung-Chi Yao, Ya-Ju Lee

**Affiliations:** 1 Department of Physics and Center for Nanotechnology, Chung Yuan Christian University, Chung-Li, 32023 Taiwan; 2 Department of Photonics, Research Center Energy Technology and Strategy, National Cheng Kung University, Tainan, 701 Taiwan; 3 Department of Photonics, National Chiao Tung University, Hsin-Chu, 300 Taiwan; 4 Department of Electronic Engineering, Chung Yuan Christian University, Chung-Li, 32023 Taiwan; 5 Institute of Electro-Optical Science and Technology, National Taiwan Normal University, Taipei, 116 Taiwan

## Abstract

Graphene quantum dots (GQDs) with an average diameter of 3.5 nm were prepared via pulsed laser ablation. The synthesized GQDs can improve the optical and electrical properties of InGaN/InAlGaN UV light emitting diodes (LEDs) remarkably. An enhancement of electroluminescence and a decrease of series resistance of LEDs were observed after incorporation of GQDs on the LED surface. As the GQD concentration is increased, the emitted light (series resistance) in the LED increases (decreases) accordingly. The light output power achieved a maximum increase as high as 71% after introducing GQDs with the concentration of 0.9 mg/ml. The improved performance of LEDs after the introduction of GQDs is explained by the photon recycling through the light extraction from the waveguide mode and the carrier transfer from GQDs to the active layer.

## Introduction

GaN-based light emitting diodes (LEDs) using quantum well structures have been implemented in the ultraviolet (UV) range over the past decades^1–3^. Such LEDs have attracted considerable attention due to their wide varieties of applications such as sterilization, water/air purification, and medical devices^4, 5^. To further increase the optical output power, it is essential to overcome several drawbacks in the GaN-based UV LEDs including low material quality in AlGaN layer, incomplete current spreading, high optical absorption in the p-GaN clad/contact layers, and high current-induced degradation. Several strategies have been proposed to improve the light output of III-nitride based UV LEDs^6–8^. For example, novel geometrical designs such as the quantum-dot and photonic-crystal structures have been suggested to confine the carriers or photons in nanostructures and enhance the light extraction efficiency (LEE) in UV LEDs^6, 7^. In addition, an electrochemical potentiostatic activation method has led to the activation of p-type materials, which is beneficial for improvement of the internal quantum efficiency (IQE)^8^. The transparent and current spreading electrode materials of GaN-based LEDs have also been investigated^4, 9, 10^. AuCl_3_-doped graphene or graphene combined with Ag nanowires (Au nanoclusters) has been considered a replacement to indium tin oxide (ITO) for transparent and conductive electrodes^4, 9, 10^. A decrease in sheet resistance and an increase of optical transmittance in the UV region were implemented using the above graphene-related electrodes.

Graphene quantum dots (GQDs) are a new class of graphene nanostructures, which consist of few layered graphene with lateral nano-sized dimensions. Differing from the zero-bandgap semimetallic graphene, GQDs create discrete bandgaps due to quantum confinement and reveal a variety of fascinating properties such as high photoluminescence (PL), chemical stability, low toxicity, and biocompatibility. GQDs have shown great potential in improving the performance of optoelectronic devices such as solar cells and LEDs^11–16^. Recently, GQDs have been used as electron acceptors, solar harvesting materials, and intermediate buffer layers in solar cells, wherein GQDs facilitate the charge carrier transport and enhance the cell efficiency^13, 14^. Due to their luminescence properties GQDs have been used for developing the light emitting layer in organic LEDs (OLEDs) or replacing phosphors in white light emitting diodes (WLEDs). The former has been implemented by mixing of methylene blue functionalized GQDs (MB-GQDs) and poly(2-methoxy-5-(2-ethylhexyloxy)-1,4-phenylenevinylene) (MEH-PPV), which leads to a decrease of the turn-on voltage by 33% compared to the OLED with pure MEH-PPV^15^. On the other hand, by introducing GQD-agar composites onto blue emitting LEDs as color converters, the GQD-related LEDs result in a luminous efficiency and light conversion efficiency of 42.2 lmW^−1^ and 61.1%, respectively^16^.

In III-nitride LEDs, a lot of photons emit outside the escape cone due to the large value of the refractive index in GaN-based materials, leading to a loss of emitted light and a reduction of light extraction. Photon recycling is an effect describing a re-capture of the photons propagating outside an escape cone and re-emission of photons from the active layer into the escape cone, enhancing the extraction efficiency in LEDs^17^. Photon recycling effect is usually unapparent in the quantum-well (QW) based LEDs because of low optical absorption in the thin QW layer^18^. This effect could be enhanced through an interaction between a high-absorption material and an evanescent field from optical cavity modes. GQDs provide high optical absorption in the UV region and they can be a candidate for improving the photon recycling in UV GaN-based devices. In this paper, we study the influence of the LED performance in InGaN/InAlGaN UV LEDs after introduction of GQDs. Such GQD-LED composites were characterized by the current-voltage (I-V) characteristics, light output-current (L-I) characteristics, and electroluminescence (EL). It was found the light output power (series resistance) in the GQD-LED composite exhibited a pronounced enhancement (reduction) compared to the LED without GQDs. Time-resolved PL and optical reflectance spectra have been used to investigate the light enhancement from the GQD-LED composite. The improvement of electrical and optical properties in the GQD-LED composite is explained by photon recycling from the waveguide modes.

## Materials and Methods

### Fabrication of InGaN/InAlGaN UV LEDs

**Figure 1:**
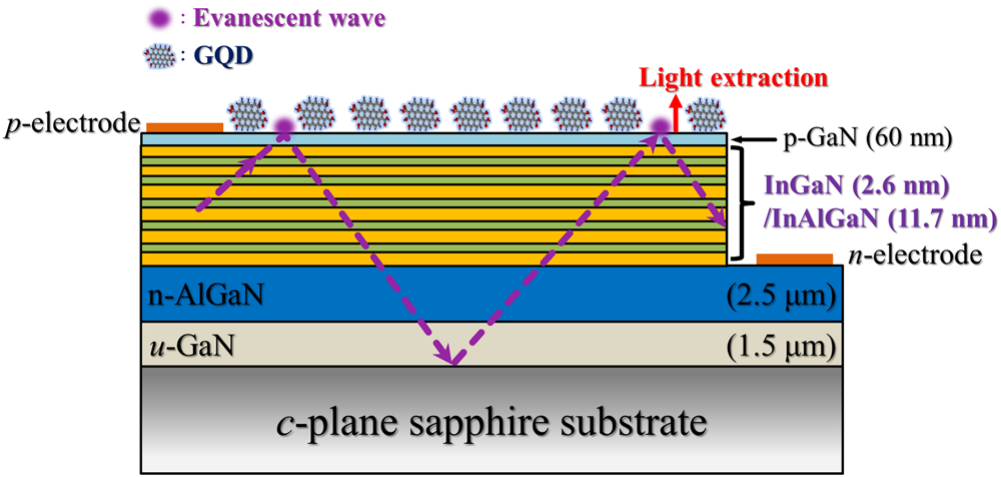
Schematic cross section of the InGaN/InAlGaN UV LEDs. The direction of light extraction was displayed by an arrow.

The InGaN/InAlGaN UV LEDs employed in this study were grown on c-plane sapphire substrates with atmospheric-pressure metal organic chemical vapor deposition system. A nucleation GaN layer was first deposited on the substrate, followed by a 1-μm-thick undoped GaN and a 2.5-μm-thick n-type Al_0.02_Ga_0.98_N. The InGaN/InAlGaN multiple-QW active layers consist of 5 periods of In_0.025_Ga_0.975_N QW layers that are ~2.6 nm thick and In_0.0085_Al_0.1112_Ga_0.8803_N barrier layers that are ~11.7 nm thick. Subsequently, the multiple QWs were capped with a 15-nm-thick p-Al_0.3_Ga_0.7_N layer and a 10-nm-thick p-Al_0.1_Ga_0.9_N layer, followed by a 60-nm-thick p-doped GaN contact layer. Figure 1 shows a schematic of the InGaN/InAlGaN UV LEDs investigated in this study.

### Synthesis of GQDs

The GQDs investigated were prepared according to a pulsed laser ablation method^19^. In brief, a 0.03 g of graphene powders was added to 600-µl methanol and mixed using a vortex mixer. The solution was allocated on a rotating stage with an angular velocity of 80 rpm. Laser ablation was performed in the solution for 5 min using an optical parametric oscillator (OPO) laser with an energy density of 2.58 J/cm^2^, a duration of 10 ns, a wavelength of 415 nm, and a repetition rate of 10 Hz. After laser irradiation, the resulting products were centrifuged, followed by filtering through a 0.22 μm syringe filters.

### Characterizations

The morphologies of the as-synthesized GQDs were examined by a transmission electron microscopy (TEM). Steady-state and time-resolved PL were carried out to investigate the luminescence properties of InGaN/InAlGaN multiple-QW active layers in LEDs. The excitation sources in PL were used by a solid-state pulsed laser with a wavelength of 260 nm, a repetition frequency of 20 MHz, and a duration of 250 fs. The collected luminescence was dispersed by a 0.75 m spectrometer and detected with a high-speed photomultiplier tube (PMT). The time-resolved PL was performed using the technique of time-correlated single-photon counting (TCSPC). The instrument response of the time-correlated single photon counting system is around 250 ps.

The as-synthesized GQDs were deposited onto the LED by drop casting a solution of GQDs, followed by drying at room temperature. The drop casted GQDs were characterized by atomic force microscope (AFM) (PSIA XE-100) to study the morphology on the LED surface. I–V characteristics of LEDs were measured with a source meter (Keithley-2400). In the EL spectrum measurements, the output EL intensity from the top side was collected and dispersed by a 0.75 m spectrometer and detected with a photomultiplier tube (PMT). The light output power and external quantum efficiency (EQE) were measured by using a calibrated integrating sphere. The reflectance spectrum was measured using an UV/vis spectrophotometer in a wavelength range of 300–900 nm.

## Results and Discussion

**Figure 2:**
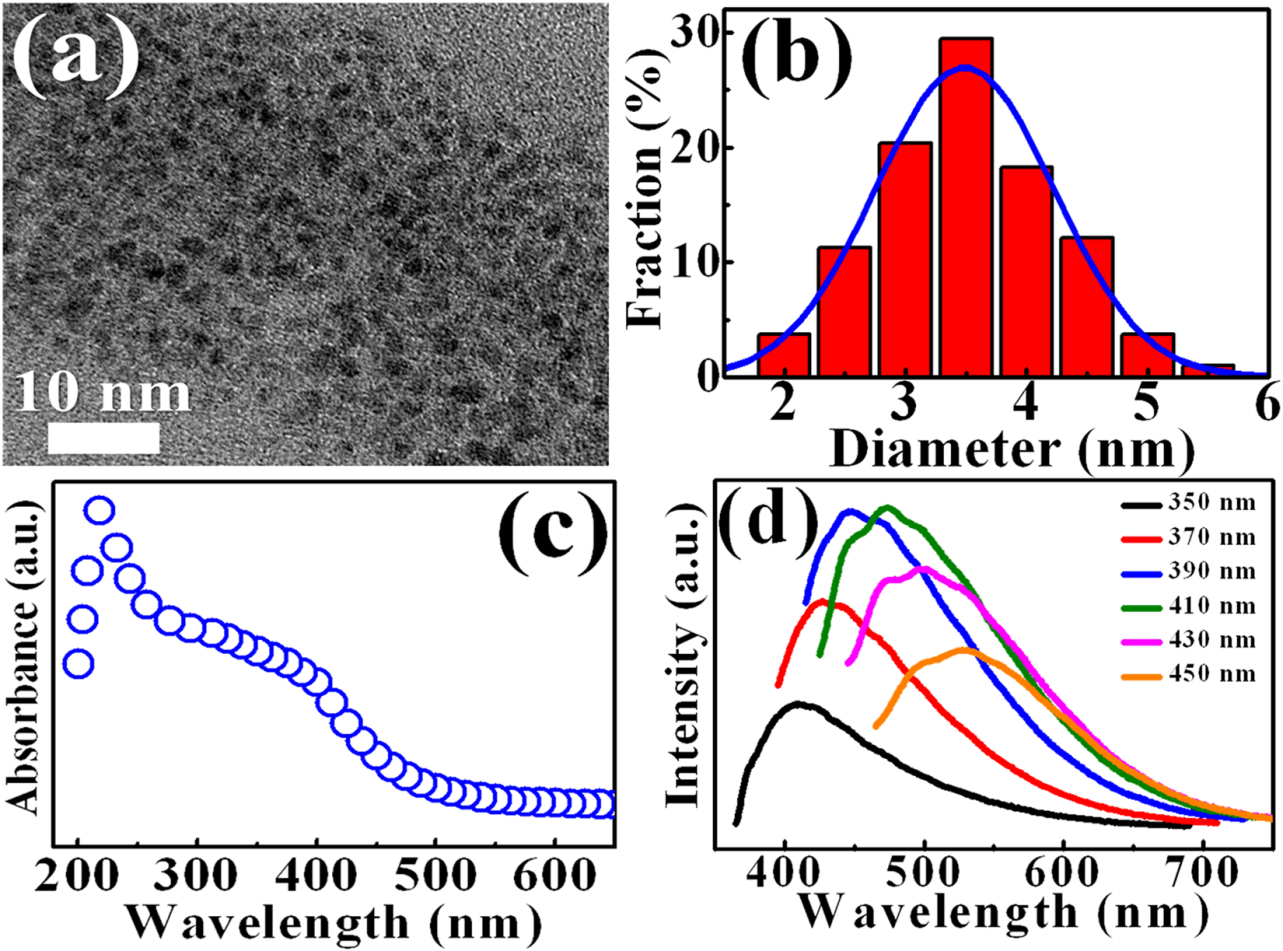
(**a**) High-resolution transmission electron microscopy (HRTEM) image for the graphene quantum dots (GQDs). (**b**) Histogram and Gaussian distribution of the GQD sizes. (**c**) The UV-Vis absorption spectrum of GQDs. (**d**) Photoluminescence spectra of the as-synthesized GQDs under different excitation wavelengths (350–450 nm).

**Figure 3:**
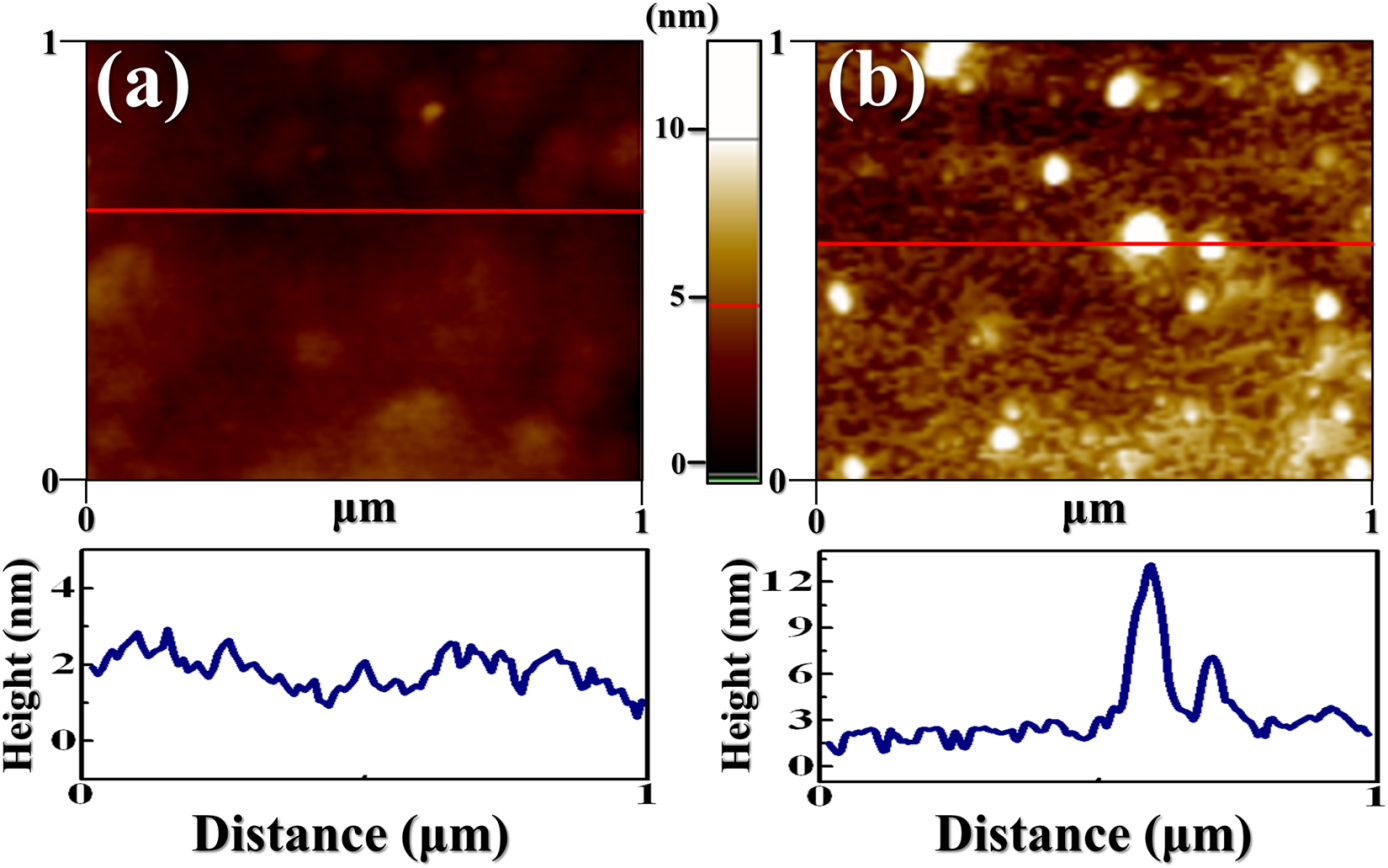
AFM images of the LED surface without (**a**) and with (**b**) the deposition of GQDs. The height profile along the line are shown below the image.

**Figure 4:**
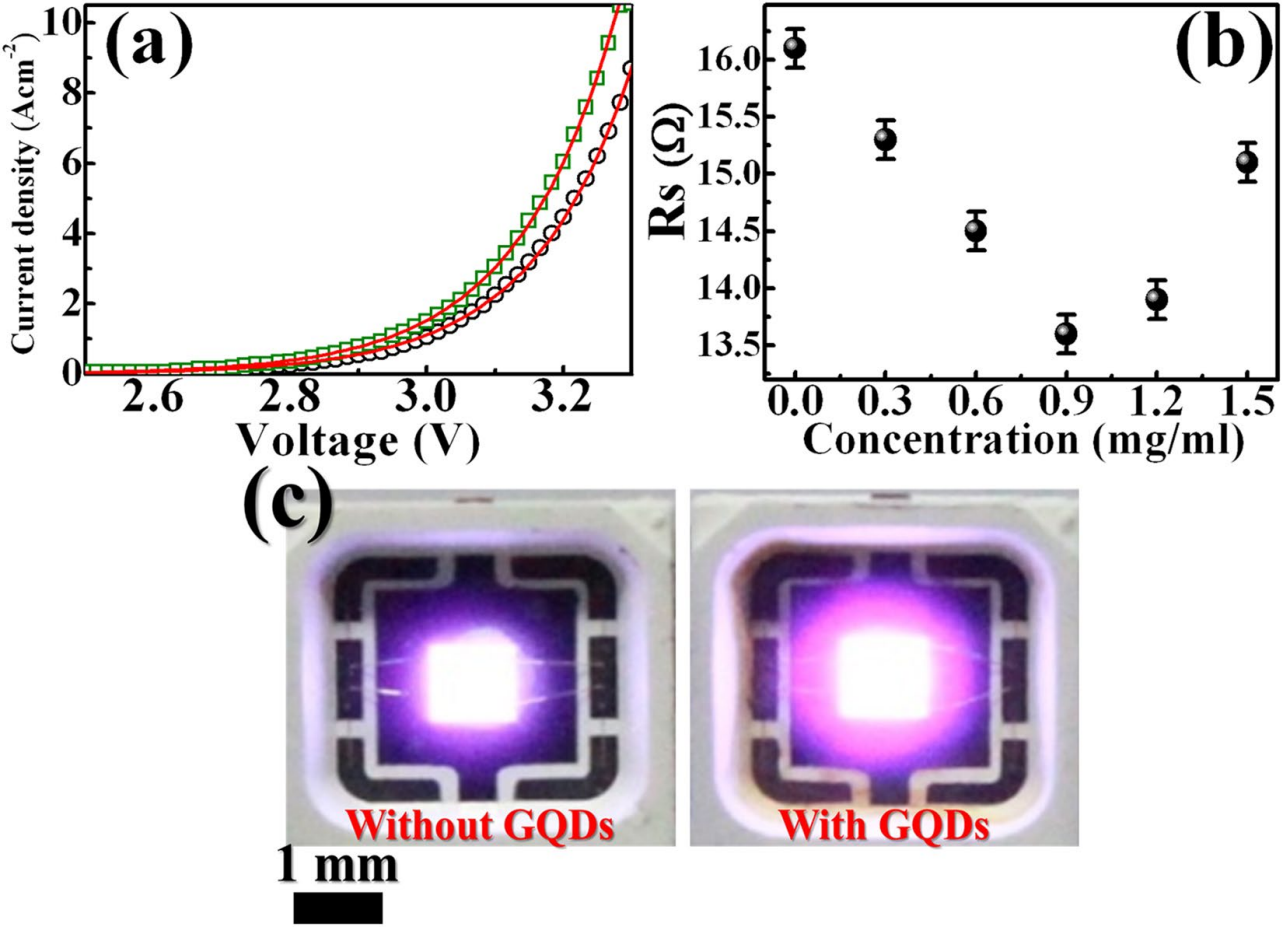
(**a**) *I*–*V* characteristics of the InGaN/InAlGaN LEDs without (open circles) and with (open squares) deposition of the GQDs. The solid lines display the calculated current using Eq. (1). (**b**) Series resistance for the GQD-deposited InGaN/InAlGaN LED as a function the GQD concentration. (**c**) Optical micrographs of the InGaN/InAlGaN LEDs without (left) and with (right) deposition of GQDs under an injection current of 100 mA.

Figure 2a shows the TEM image of the synthesized GQDs deposited on an electron microscope grid from the GQD solution. The statistics of the size distribution as determined from different regions indicate an average size of 3.5 ± 0.1 nm, displaying in Fig. 2b. Figure 2c displays the optical UV-Vis absorption spectrum of the GQDs. The optical absorption is dominated by a peak at ~230 nm, which is typically assigned as the π-π* plasmon transitions of aromatic sp^2^ domains^20, 21^. A shoulder appeared at 380 nm has been associated with the GQD structures^20, 21^. The GQDs shows relatively strong absorption in the UV region, which is advantageous for the photon recycling effect in the InGaN/InAlGaN UV LEDs. Figure 2d shows PL spectra of the GQDs by using different excitation wavelengths. The PL peaks of GQDs shift with changing excitation wavelengths from 350 to 450 nm. The excitation-dependent PL behavior involving GQDs has been observed in previous studies, originating from different emitting species or the localization of electron-hole pairs^22, 23^. To determine the surface morphology and the GQD thickness on LED surface, AFM measurements were performed. Figure 3a and b display AFM images of the LED surface without and with the introduction of GQDs, respectively. Obviously, additional white spots appeared in Fig. 3b represent the GQDs. The average height of GQDs on the LED surface are about 6–13 nm, suggesting that the GQDs typically consist of 12–26 graphene layers^24^. The I-V characteristics of the LEDs without (open circles) and with (open squares) GQDs are compared in Fig. 4a. It was found the current level at an injection voltage of 3.25 V was enhanced from 70 to 90 mA in the presence of GQDs (concentration = 0.9 mg/ml). The series resistance of LEDs, an important factor that determines the quality of the LED performance, can be obtained from the slope of the I-V curves. To estimate the series resistance, the experimental I-V characteristics were fitted by the diode equation^25^:

1 $$I={I}_{0}[\exp (q(V-I{R}_{S})/nkT)-1],$$

where *R*_*s*_ is the series resistance, *n* is the ideality factor, and *k* is the Boltzmann constant. Using Eq. 1, the I-V curves of LEDs can be fitted and the series resistance is obtained as shown in Fig. 4b. From the fits, *R*_*s*_ of the LED in the presence of the GQDs was found to decrease from 16.1 to 13.6 Ω by increasing the concentration of GQDs from 0 to 0.9 mg/ml, corresponding to a decrease in series resistance of 15.5%. A lower series resistance is beneficial in LED performance, expecting to spread current more uniformly and increase the emitting area. Figure 4c shows the EL images of the investigated LEDs in the presence and absence of GQDs, respectively, indicating the current spreading ability of the former is better than that of the latter. When the GQDs on the LED surface are irradiated by EL, the photogenerated holes from GQDs are generated and transferred into the active layer of LEDs through the top p-GaN layer (will be described later). This carrier (hole) transfer effect results in a spread of the hole carriers laterally and thus contribute to the current spreading in LEDs. Thus, the series resistance (current spreading) of the LED will be reduced (enhanced) after the introduction of GQDs.

**Figure 5:**
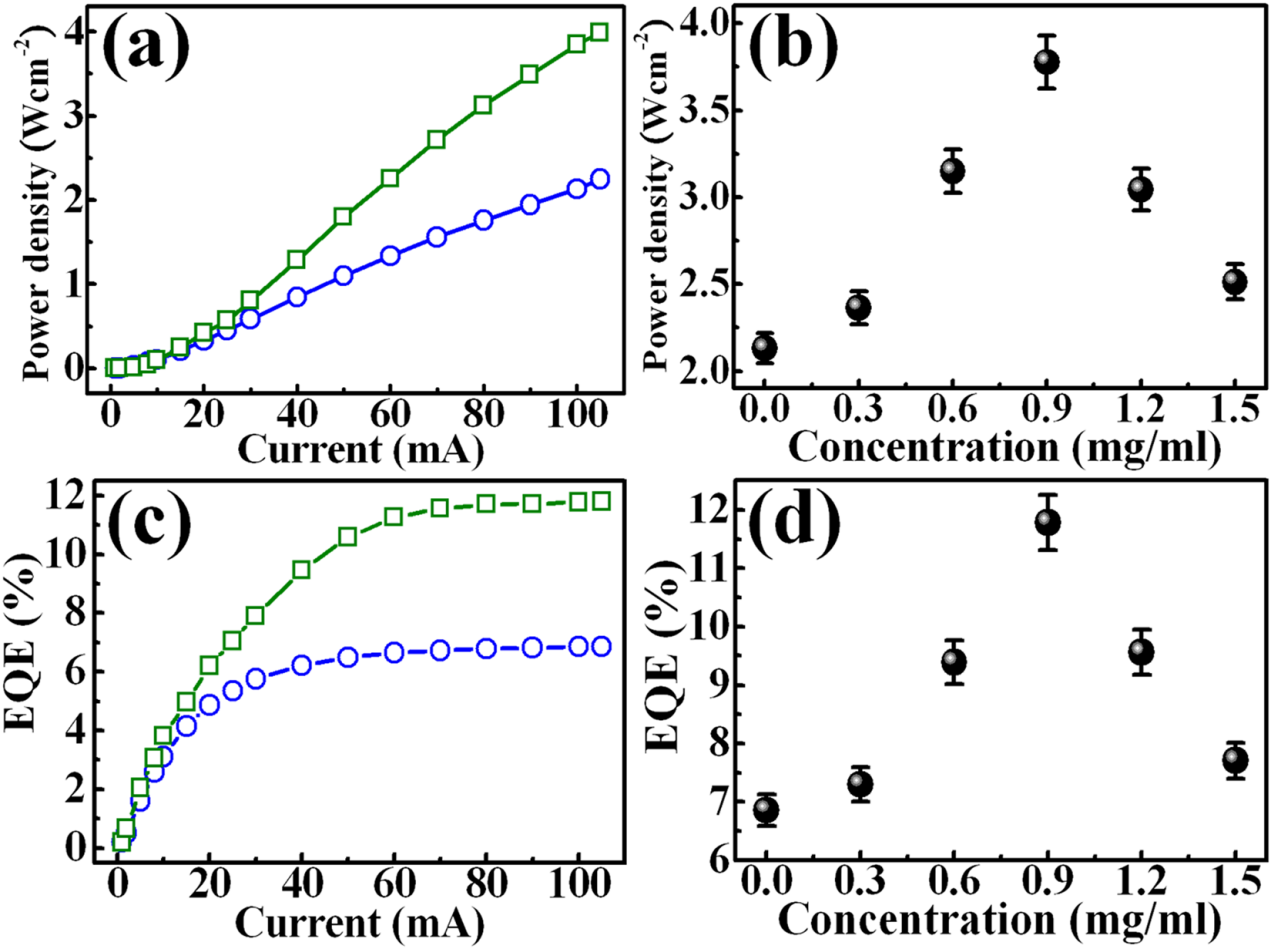
(**a**) Light-current characteristics of the InGaN/InAlGaN LEDs without (open circles) and with (open squares) deposition of the GQDs. (**b**) EL intensity of the GQD-deposited InGaN/InAlGaN LED as a function the GQD concentration. (**c**) Injection current dependence of external quantum efficiency for the InGaN/InAlGaN LEDs without (open circles) and with (open squares) deposition of the GQDs. (**d**) GQD concentration dependence of the external qunatum efficiency in the GQD-deposited InGaN/InAlGaN LED.

The introduction of GQDs on LEDs also produced a change in light emission. Figure 5a shows the light output from LEDs without and with GQDs (0.9 mg/ml) as a function of the injection current. Apparently, the light output from the LEDs with GQDs is higher than that from the LEDs without GQDs. Figure 5b shows the light output power of the LEDs with incorporation of GQDs as a function of the GQD concentration for the injection current fixed at 100 mA. The light output has a maximum value at the GQD concentration of 0.9 mg/ml, the light output power saturates and decreases after that concentration. The decrease of the light output after GQD concentration of 0.9 mg/ml is probably due to the shielding or thickening of the GQD layer on top of LEDs, blocking the emitted light from the LEDs. The EQE of the LEDs without and with GQDs at different injection currents were shown in Fig. 5c. At the applied current of 100 mA, the EQE was enhanced from 6.9% to 11.8% at the GQD concentration of 0.9 mg/ml, corresponding to an increase of the EQE by 71%. To our knowledge, the 71% increase in EQE is much better than the previous report on the enhancement of GaN-based LEDs by introducing other nanoparticles^26, 27^. Figure 5d shows the EQE of the LED as a function of the GQD concentration. In LED physics, EQE is defined as the product of the injection efficiency, the IQE, and the LEE, where the injection efficiency is usually regarded as equal to unity^3, 17, 28^. In general, IQE is basically dependent on the material quality of epilayers in LEDs, while LEE is related to structural factors such as chip geometry and surface texture^29, 30^. Because the material quality of the active layers in LEDs does not change after the deposition of GQDs on the LED surface, the enhancement of EQE should be largely dependent on LEE mostly.

**Figure 6:**
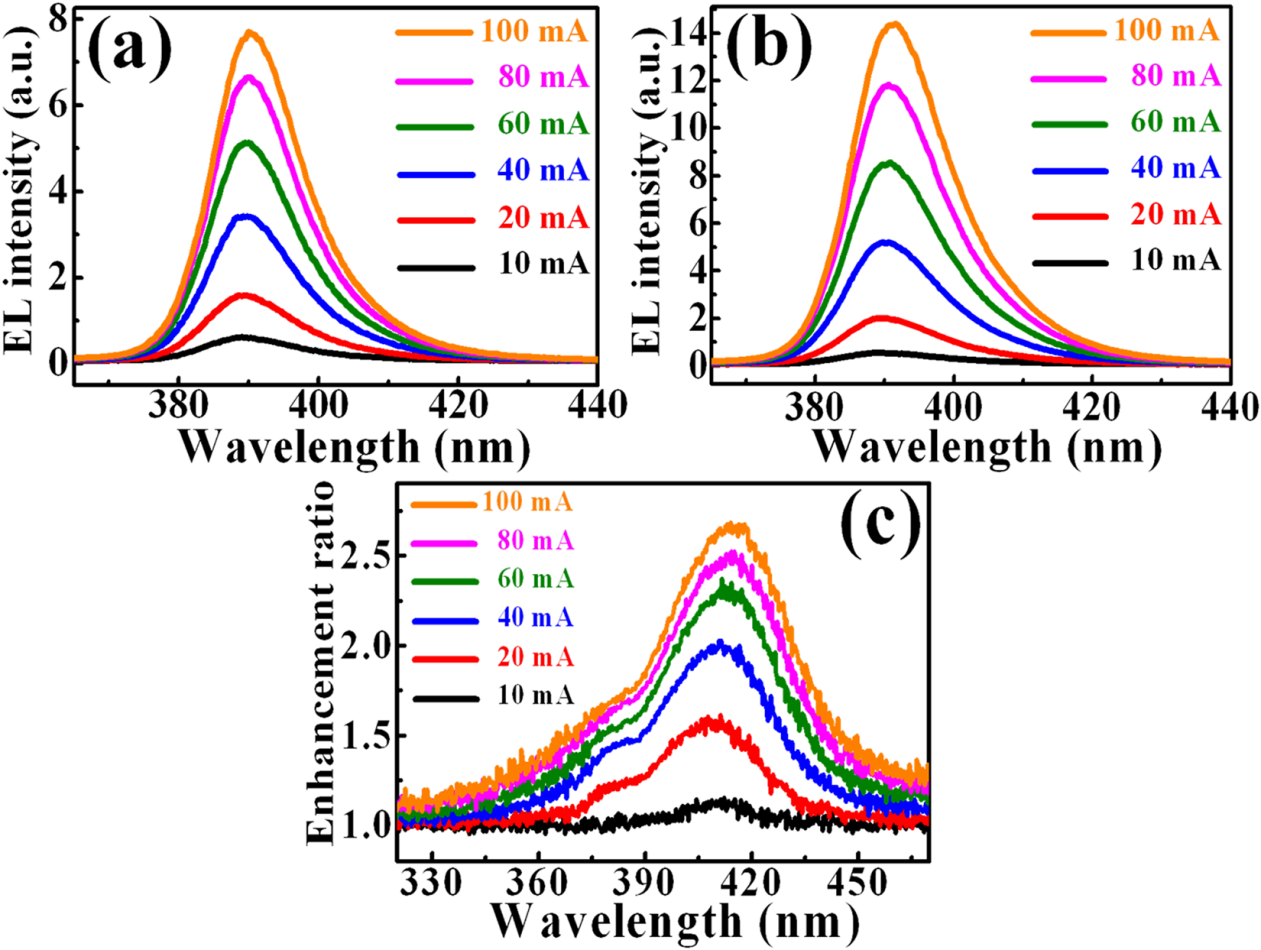
EL spectra for the InGaN/InAlGaN LEDs without (**a**) and with (**b**) the deposition of GQDs under different injection currents. (**c**) EL enhancement ratio of introducing GQDs on InGaN/InAlGaN LEDs under different injection currents.

**Figure 7:**
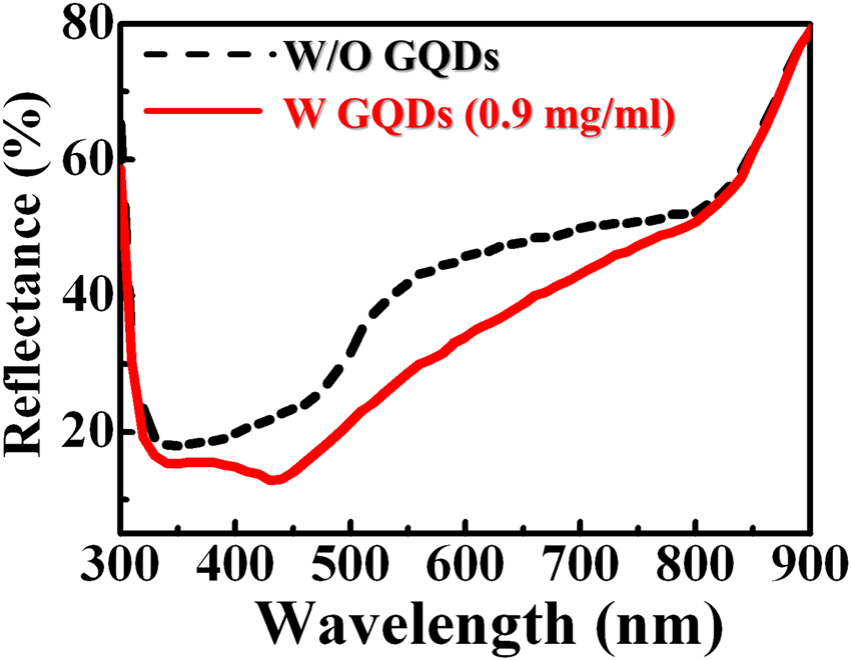
Reflectance spectra of the InGaN/InAlGaN LEDs without (dashed line) and with (solid line) the deposition of GQDs.

Figure 6a and b show the EL spectra of LEDs in the absence and presence of GQDs (0.9 mg/ml) with different injection currents, respectively. Like the result of L-I characteristics in Fig. 5a, the EL of the LEDs increases after the introduction of GQDs. There is a main EL peak at around 387 nm for the investigated LEDs, corresponding to the emission from the InGaN/InAlGaN QW in the active layer. Figure 6c shows the EL enhancement ratio (I/I_0_, where I and I_0_ are the EL intensity of the LEDs in the presence and absence of GQDs, respectively) as a function of the emission wavelength for different injection currents. The EL enhancement ratio shows a maximum value around the wavelength of 415 nm, indicating 28 nm longer than the wavelength of maximum EL in the bare LED. To find out the wavelength shift of the maximum EL in LEDs after the introduction of GQDs, the reflectance spectrum from the LED surface was measured. Figure 7 shows reflectance spectra of the LEDs without (dashed line) and with (solid line) GQDs. The reflectance of LEDs reduces pronouncedly after the introduction of GQDs, probably originating from the increase of roughness on the LED surface^31, 32^. It is noted that the wavelength of the minimum reflectance is located at ~350 and ~437 nm for the bare LEDs and the GQD-deposited LEDs, respectively. The red shift of the minimum reflectance can be used to account for the above EL enhancement ratio. With the introduction of GQDs, the EL with longer wavelengths coincides with less reflectance at the LED surface. This leads to a shift of the emitted EL toward the longer wavelength and explains the the maximum wavelength for the EL enhancement ratio (Fig. 6).

**Figure 8:**
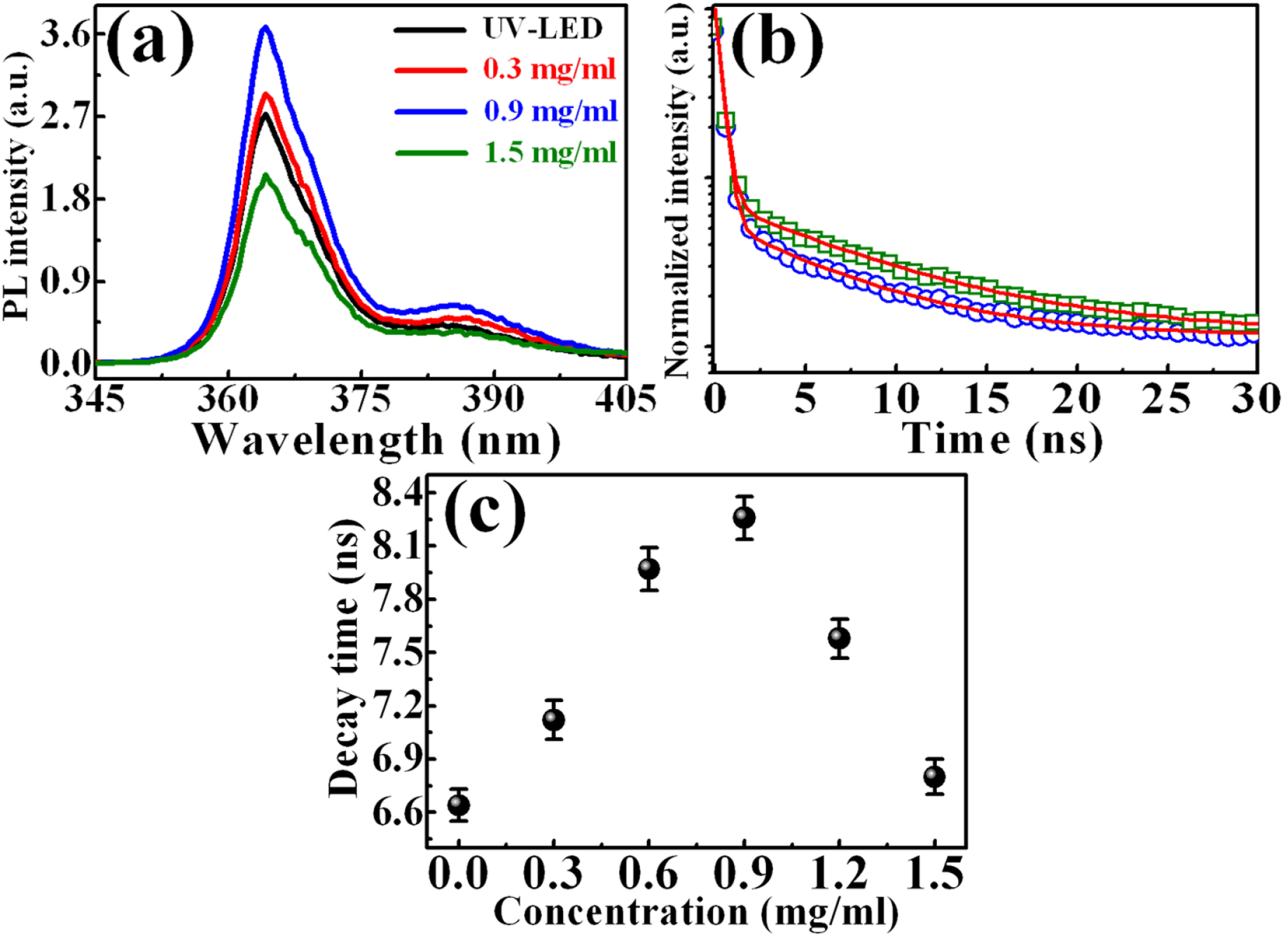
(**a**) PL spectra of the GQD-deposited InGaN/InAlGaN LED as a function the GQD concentration. (**b**) PL decay transients of the InGaN QW for InGaN/InAlGaN LEDs without and with the incorporation of GQDs. (**c**) PL decay times of the InGaN QW in the GQD-deposited LED as a function the GQD concentration.

Figure 8a shows the PL spectra of the InGaN QW in LEDs after the incorporation of GQDs with different concentrations. The spectra reveal PL peaks located at around 363 and 388 nm, which are identified to GaN layers and InGaN QWs, respectively. As shown in Fig. 8a, the PL intensity in the LED increases with increasing the GQD concentration from 0 to 0.9 mg/ml and decrease after the concentration of 0.9 mg/ml. The maximum PL intensity occurs at the GQD concentration of 0.9 mg/ml, revealing an enhancement factor of 33%. The enhancement of PL intensity indicates an increase of the recombined carriers in the LEDs after introduction of GQDs. The PL decay profiles of the InGaN QW in LEDs without and with the incorporation of GQDs are displayed in Fig. 8b, indicating the carrier lifetime of the InGaN QW with GQDs is longer than that of the bare InGaN QW. The PL decay profiles can be fitted by a double exponential function $n(t)={n}_{1}(0){e}^{-t/{\tau }_{1}}+n(0){e}^{-t/{\tau }_{2}}$, where *τ*_1_ and *τ*_2_ represent the carrier lifetime of the fast and slow decay, respectively. The solid lines of Fig. 8b display the fitted results according to the double exponential function. To investigate the origins of *τ*_1_ and *τ*_2_, the PL decay transients monitored at the peak of GaN were measured (not shown). It was found that the PL decay transient of the GaN peak corresponds to *τ*_1_ quite well, indicating *τ*_1_ is originated from the carrier lifetime of GaN layers. Thus, only *τ*_2_ represents the carrier lifetime in InGaN QWs. The carrier lifetimes of InGaN QWs in the GQD/LED composite versus the GQD concentration are displayed in the solid circles of Fig. 8c. The carrier lifetime of InGaN QWs increases with increasing the GQD concentration. It reaches a maximum value of 8.3 ns with the GQD concentration of 0.9 mg/ml and begins to decrease after that concentration. The decrease of the carrier lifetime after GQD concentration of 0.9 mg/ml is probably due to the shielding or thickening of the GQD layer, reabsorbing the carriers generated from GQDs and blocking the carrier transfer from GQDs to the active layer in LEDs.

**Figure 9:**
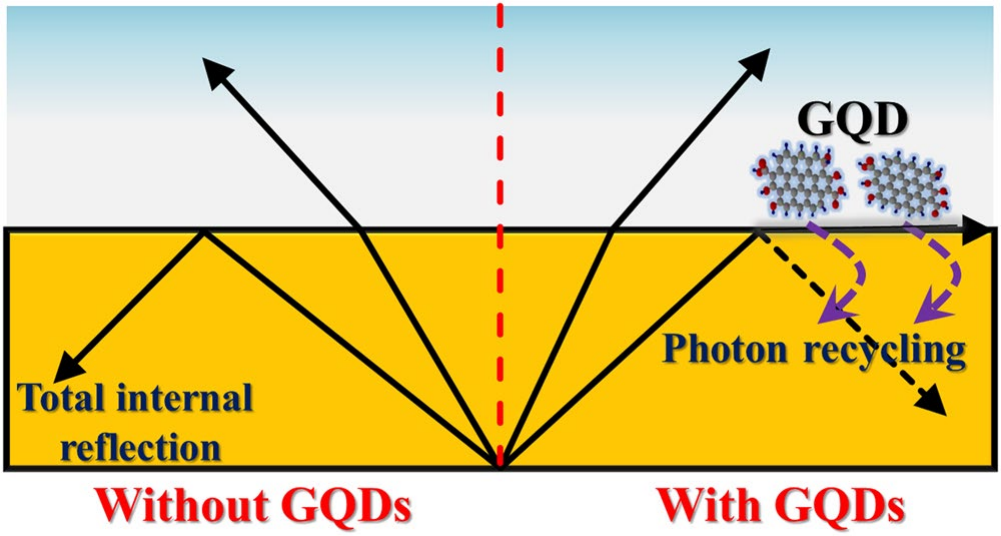
Schematic illustration of the photon recycling effect in a GQD-deposited InGaN/InAlGaN UV LED.

**Figure 10:**
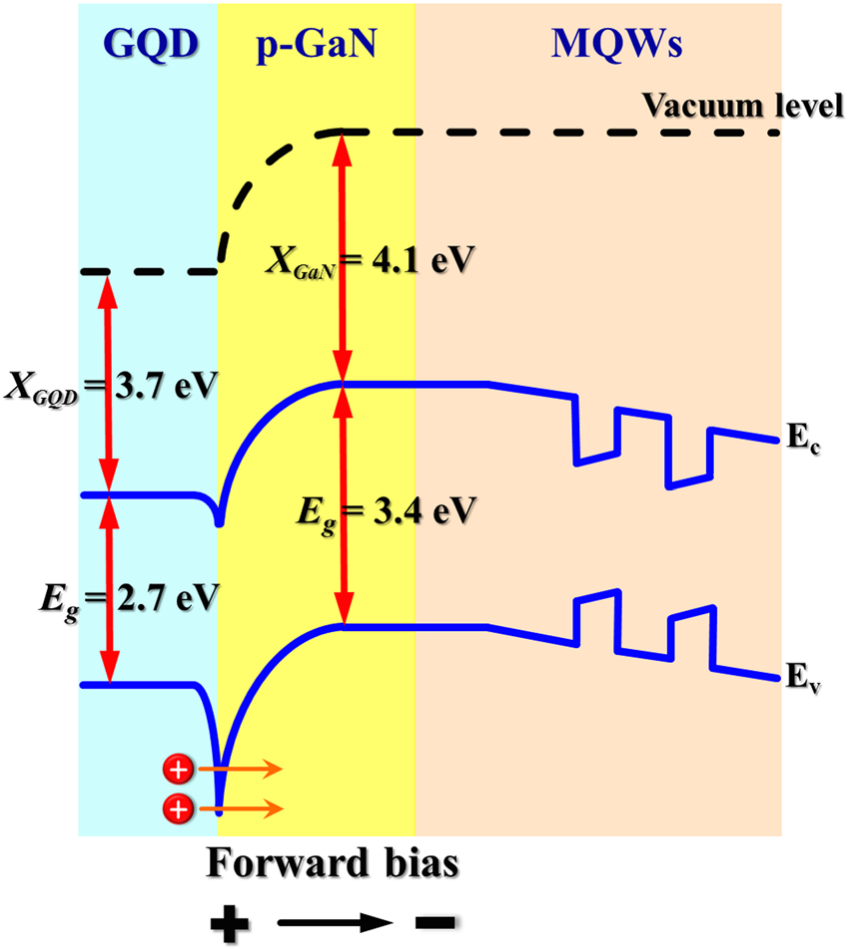
Energy band diagram of the GQD/LED interface under forward bias.

On the basis of the above observations, a photon recycling scheme was proposed for explaining the improvement of properties in LEDs after the introduction of GQDs (Fig. 9). It is well known that the photons outside the escape cone (waveguide modes) generate the evanescent field near the air/semiconductor interface due to total internal reflection. When GQDs are incorporated on the LED surface, the evanescent field interacts with GQDs and the energy of the evanescent light would be extracted by GQDs (the direction of light extraction is shown in Fig. 1). Upon absorption of light in the optical waveguide mode, electron-hole pairs from the GQD layer are generated around the surface of LEDs and separated by the forward voltage. Under applied forward bias, the generated holes are injected into the valence band of p-GaN through tunneling and moved to the InGaN QWs in the active layer (Fig. 10). The injected holes can then recombine with electrons radiatively and produce enhanced EL from LEDs. Thus, the light output power was enhanced due to the increased hole injection, stemming from the introduction of GQDs on the LED surface. In other words, the photons outside the escape cone (waveguide modes) are extracted due to the deposition of GQDs, and a number of holes from GQDs are transferred into the active layer, recycling the light emission in LEDs. The photon recycling also increases the carrier density of the InGaN QWs in the active layers, leading to an enhanced carrier lifetime in the time-resolved PL studies. With increasing the concentration of GQDs, more holes in GQDs would be generated and transferred into the InGaN QWs in the active layer, resulting to a higher carrier lifetime in InGaN QWs, as shown in Fig. 7c.

## Conclusions

GQDs with an average size of 3.5 nm have been successfully synthesized by pulse laser ablation. By incorporating the synthesized GQDs on the surface of InGaN/InAlGaN UV LEDs, both electrical and optical performances of LEDs improve remarkably. An enhancement of EL intensity by 71% and a reduction of series resistance by 15.5% for LEDs have been achieved after incorporation of GQDs with the concentration of 0.9 mg/ml. We suggest that the photon recycling, which contains the light extraction from the waveguide mode and the hole transfer from GQDs to the active layer, is responsible for the enhancement of light output in the UV LEDs with the introduction of GQDs.
